# Scenario and future prospects of microRNAs in gastric cancer: A review

**DOI:** 10.22038/ijbms.2019.32399.7765

**Published:** 2019-04

**Authors:** Showkat Ahmad Bhat, Sabhiya Majid, Muneeb U Rehman

**Affiliations:** 1 Department of Biochemistry, Govt. Medical College, Srinagar Jammu & Kashmir, India

**Keywords:** Biomarkers, Clinical applications, Diagnosis and prognosis, Gastric cancer, microRNAs, Noninvasive biomarkers

## Abstract

Carcinoma of the stomach is one of the major prevalent and principal causes of cancer-related deaths worldwide. Current advancement in technology has improved the understanding of the pathogenesis and pathology of gastric cancers (GC). But, high mortality rates, unfavorable prognosis and lack of clinical predictive biomarkers provide an impetus to investigate novel early diagnostic/prognostic markers and therapeutic targets for GC, which are sufficiently sensitive to GC. Current biomedical investigations have explored several budding GC biomarker by utilizing serum proteins, natural oncogenic genes during improvement in molecular technologies as microarray, and RNA/DNA-Seq. Recently, small non-coding microRNAs (miRNAs) are becoming vital regulators in oncogenesis pathways and can act as handy clinical biomarkers. The newly introduced class of biomarkers is rising as new molecules for cancer diagnosis and prognosis. For better understanding of the gastric carcinogenesis, miRNAs may help to elucidate the mechanisms of tumor growth and can help to discover novel untimely potent markers for early detection of GC. Here in this review, we summarize the recent research studies supporting the utility of miRNAs as novel early diagnostic/prognostic tools and therapeutic targets. Thus, here we introduce potential future treatment strategies for gastrointestinal (GI) cancers, which indicate the practicality and clinical applications of miRNAs in GC.

## Introduction

MicroRNAs (miRNAs) are a group of highly conserved small non-coding RNAs (18 to 24 nucleotides) that regulate a wide range of biological processes including carcinogenesis. In cancer cells, miRNAs have been found to be heavily dysregulated (1-4). Ambros and his team were the first ones to discover miRNA in *Caenorhabditis elegans* (*C. elegans*) in the 1993 (5). It was recognised as small non-protein coding RNA, which affects development through regulation of expression of protein lin-14. Another group lead by Reinhart in 2000 reported 2^nd^ miRNA let-7 in *C. elegans*, and was shown that negatively regulates the expression of heterochronic gene lin-41 via a sequence specific RNA-RNA interaction with the 3′-untranslated regions of its mRNA (6). Further research by three independent groups in early years of 21^st^ century demonstrated the presence of abundance of miRNAs in both vertebrates and invertebrates (7-9). Few of these miRNAs are highly conserved, suggesting that miRNA may mediate post-transcriptional regulation as usual regulatory function across the species. Cancer of stomach in current scenario is fourth mainly frequent and 2^nd^ most mortality causing cancer across the world (10-12). It can be difficult to detect stomach cancer until it reaches to advanced stages of the disease because the symptoms tend to be indistinguishable from other gastrointestinal problems. Gastric cancer (GC) has remained a main clinical challenge till date, because of its poor prognosis, inadequate treatment options, somewhat resistance to chemotherapy / radiotherapy and its late diagnosis (13). At present, tumor serum markers like carcinoembryonic antigen (CEA), *Helicobacter pylori* (*H. pylori*) antibodies, histopathology, endoscopy and assay of pepsinogen are the main tools used for diagnosis and assessing the disease, but all of these diagnostic approaches are less responsive for early detection (14-17). Current studies of molecular biology have discovered that certain gene alterations in GC tissue might be associated with some premalignant lesions. Every molecules linked with GC can be considered as a possible prediction marker of GC, even in late premalignant situations. Since there is no good early stage biomarker to efficiently diagnose GC and its corresponding stages at present, the newly introduced microRNAs (miRNAs) in addition to other early stage genetic biomarkers may act as newly dynamic budding biomarkers in the diagnosis of human disease. Therefore, miRNAs that are connected with the incidence or progression of cancers may act as early potential markers for cancer diagnosis.

***Synthesis of miRNA***

**Figure 1:**
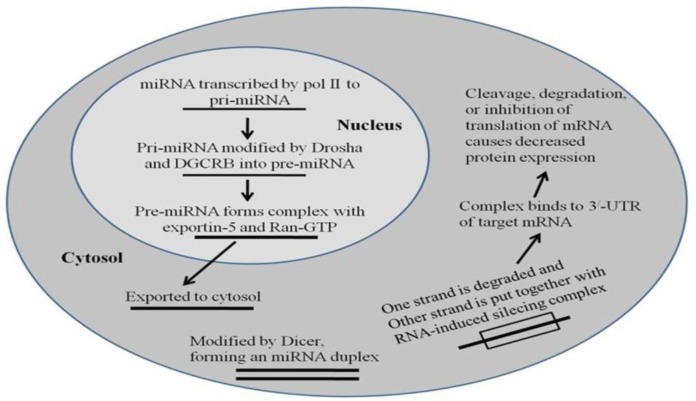
Synthesis of microRNAs within the cell

The small miRNAs are single-stranded RNA molecules and contain 20-22 nucleotides, which do not code for proteins (18). These miRNAs are transcribed from miRNA genes in the presence of RNA polymerase II and III, forming primary miRNAs, or pri-miRNAs and are then cleaved by Drosha enzyme to create precursor miRNAs, or pre-miRNAs (19, 20). Pre-miRNA that is a hairpin like structure is cleaved once transported into cytoplasm to generate a miRNA duplex by a protein called Dicer to give final mature miRNA, which can dictate cellular events (21, 13). The less stable strand from the miRNA duplex is typically added to another protein, RISC (miRNA Induced Silencing Complex), whose formation is induced by Dicer, where it can have other effects on the target gene in terms of its protein expression (Figure 1). These effects are most often observed when one strand of miRNA was linked to the 3’-untranslated area (UTA) of the mRNA sequence (23, 24).

***Tumorigenesis and miRNAs***

The role of miRNA in tumorigenesis is emphasized by the association of cancers with genomic alterations, which can potentially deregulate their repression. Changes in miRNA expression are expected to influence the activities of targeted mRNA encoding proteins that are oncogenic or anti-oncogenic function. When gene expression profiles are used to compare cancerous and normal tissues, it has been found that miRNAs and also mRNAs are deregulated (25, 26). This information may be used to infer that tumorigenesis comes from a change within the collection of miRNAs in the genome (miRNome). In addition to above, it has been found that certain miRNAs are deregulated more often than others, which suggests that they are playing a key role in tumorigenesis (27). In the beginnings of miRNA research, miRNAs were believed to have similar effects on gene expression (i.e. negative regulation of target mRNA), but recent researches have shown that miRNAs can either repress or activate, depending on the conditions of the cell as it is believed that microRNAs do not function by themselves, through miRNPs (ribonucleoproteins) effector complexes. These miRNPs are able to gather enzymes and factors that can cleave mRNA and degrade the enzymes that further process mRNA and leads to cancer (28, 29). Also, studies have revealed and validated the role of some miRNAs in oncogenesis in animal models (31). 

Numerous studies have demonstrated an association of altered miRNA expression and cancer. An uneven number of genes encoding miRNAs are found in regions where regulation of miRNA expression can likely be disrupted by chromosomal abnormalities (32). Approximately, more than one-half of the 200 studied miRNA genes are found in cancer-associated genomic regions, which include minimal regions of amplification, minimal regions of loss of heterozygosity, and not so common chromosomal breakpoint regions (31). Exhaustive expression analysis of abnormal chromosomal regions having miRNAs has revealed intense correlation between alteration in DNA copy number and miRNA expression, suggesting that alterations in genome, principally deletion or amplification, can be a main mechanism of activation of oncogenic function of miRNA or inactivation of its tumor suppressor function (33). Since miRNAs are usually negative regulators of gene expression, changes in the amounts of these RNAs can be tumorigenic if they target mRNAs for either a tumor suppressor or an oncogene. For instance, excessive build up of a miRNA that targets the mRNA of a tumor suppressor would result in the loss of protective function. In contrast, decreased accumulation of a miRNA that targets the mRNA of a proto-oncogene could lead to accumulation of large amount of the oncogenic protein. The imbalance in the activities of tumor suppressor genes and oncogenes is the final outcome of both pathways (34, 35).

***miRNAs as genetic indicators of cancer***

In the past, oncogenes and cellular genes that regulate cellular proliferation and growth in a negative fashion (tumor-suppressor genes) were considered as the main genetic indicators of cancer, but recent studies suggested that miRNAs are the main genetic indicators of cancer; the miRNAs concerned with carcinogenesis are called oncomirs (36, 37). It has been reported that 50% of genes encoded by miRNAs are located at certain sites called fragile sites where chromosomal rearrangements associated with cancer often occur (38). Yet, in most cancers, miRNAs are seemingly deregulated, which may be caused by transcriptional deregulation, epigenetic alterations (DNA methylation, mutation, and DNA copy abnormalities) and problems in miRNA biogenesis pathways; these mechanisms can either work alone or together in order to deregulate miRNAs. Certain families of miRNAs regulate cell-cycle and cell-cycle exit (senescence) in addition to cell differentiation and proliferation and, if mutated, can cause abnormalities in the cells. The mutation in any given miRNA of a somatic cell can lead to tumorigenesis and if are present in the germ line cells it may be precursor to cancer (39-43).

**miRNAs and its role in gastric cancer as a noval diagnostic and prognostic biomarker**

**Figure 2:**
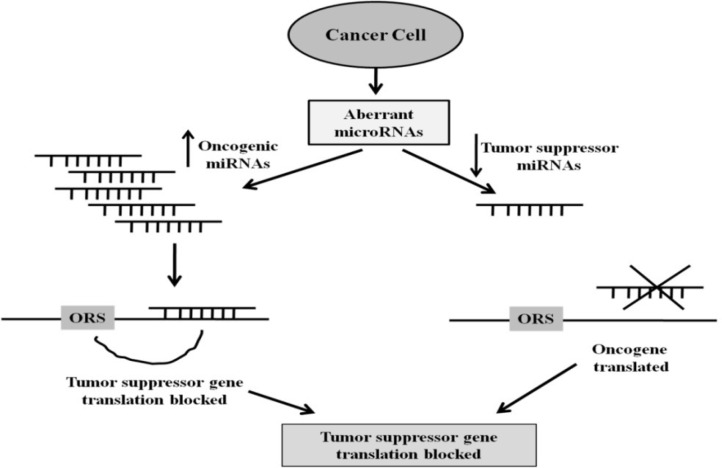
The role of microRNAs (miRNAs) in tumor formation/creation

As in current scenario due to the poor prognosis, inadequate treatment options, relative resistance to radiotherapy / chemotherapy, and late diagnosis, detection of GC biomarkers remained a major clinical challenge for researchers. Hence, longstanding target of GC research was to recognize specific and reliable methods for early diagnosis and management of cancer. Over the last past years, the scientists have begun to investigate the possible utility of the miRNAs as early and specific biomarkers, among these some are concerned in GC tumorigenesis, proliferation, invasion and metastasis (44, 45). It is shown that miRNAs are the latest leading group of non-protein coding RNA molecules that do their function by base pairing among the seed region of miRNA and 3′-UTA of target gene. Dysregulated miRNAs can take part either as tumour-suppressive or an oncogene in regulating cell growth, cell cycles and cell migration, depending on their target genes within GC as shown in Figure 2 (46, 47). There are several circulating miRNAs present in body fluids (plasma, sera, tears, urine, amniotic fluid and gastric juice). Circulating miRNAs showed different expression patterns in body fluids that might be due to different cell types under certain physiological conditions (46). According to aforementioned, miRNA have the capacity to be used as useful non-invasive biomarker for diagnosis of cancer.

Genome studies (48, 49) have shown that miRNA genes are regularly situated inside the regions of the loss of heterozygosity, amplification, fragile sites and other cancer-associated genomic regions, which suggests the vital role of miRNAs in tumorigenesis (50). Additional studies have revealed that upregulated and downregulated miRNAs might have important role in tumorigenesis as a new broad-spectrum oncogenes and tumor suppressor genes in GC (51). Meanwhile, other miRNAs are reported to be down or upregulated in plasma/serum/tissue of GC patients as summarized in Table 1.

***Experimental-based evidences of miRNAs as diagnostic and prognostic biomarker for gastric cancer***

There are some miRNAs that have shown positive associations with the GC, indicating that miRNAs can act as diagnostic and prognostic biomarkers for GC in future (a): miR-372 having oncogenic character in controlling cell growth, cell cycle and apoptosis via down-regulation of LATS2 tumor suppressor gene (52). (b): The proliferation and development of GC cancer cells has shown positive relation with the over-expression of miR-650 at least partly through directly targeting the ING4gene (53). (c): The down-regulation of mir-663 in tumor cells may lead to development of the GC, in association with the hyperplasia of aberrant cells (54). (d): Some studies showed that the aberrant over-expression of miR-126 and consequent SOX2 down-regulation might contribute to gastric carcinogenesis (55). (e): Nuclear factor kappa B 1 (NF-kappaB1) may be targeted by miR-21, miR-16 and miR-9 and can regulate growth within GC cells, which suggests a remarkable tumor suppressive activity in the gastric pathogenesis (56). (f): There is a lot of evidence that cholecystokinin B receptor (CCKBR) was targeted by miR-148b and suppressed significantly the growth of GC cells. (57). (g): The inhibitory effect of miR-141 and miR-451 on cell proliferation can be involved in the developmental progression of GC (58-60). (h): Family of miR-29 and the ectopic over-expression of miR-101 may be apparently slow down the cell growth, migration, and invasion of the GC cells via targeting the Mcl-1 Fos, Cdc42 EZH2 and cyclooxygenase-2 (Cox-2) genes, respectively. (61, 62). (i): Different studies have shown that miR-10b, miR-21, miR 126, miR-223, miR-30a-5p and miR-338 are directly and extensively related with relapse-free and as a whole survival in GC patients (63-66).

**Figure 3:**
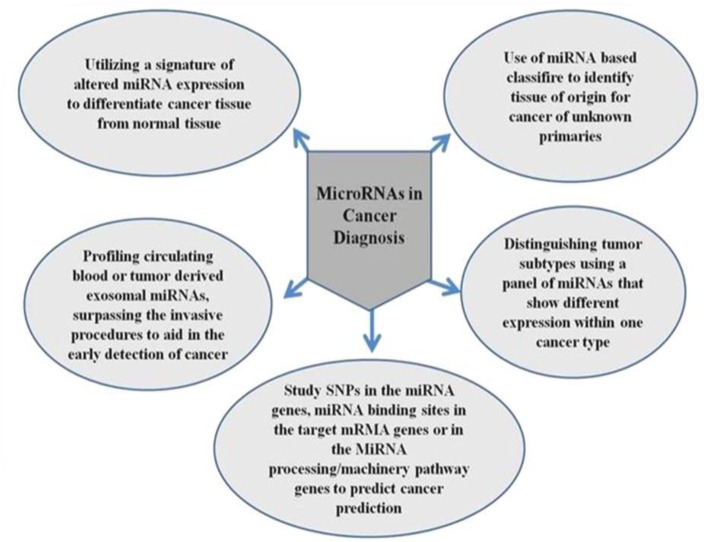
MicroRNAs (miRNAs) in different disease diagnosis

There are also different aspects of miRNAs, which make available noval ways of utilizing miRNAs in the diagnosis of disease as in Figure 3.

**Past and current scenario of miRNAs in cancer**

Several circulating miRNAs in the blood of GC patients can be practically used as non-invasive diagnostic and prognostic biomarkers, including let-7a, miR-1, miR-17-5p, miR-196a, miR-20a, miR-21, miR-27a, miR-34, miR-106a/b, miR-199a-3p, miR-218, miR-221, miR 223, miR- 370, miR-376c, miR-378, miR-421, miR-423-5p, miR-451 and miR-486 (46, 82-84). The patterns of expression of some miRNAs including miR-221, miR-744 and miR-376c in the serum may be used as population screening biomarkers to differentiate among gastric cancerous and healthy individual (46, 84). 

In recent conducted studies, it has been shown that expression patterns of miR-21, miR-106a and miR- 421 in gastric juice samples were significantly different within GC patients and patients with benign gastric ulcers. Hence, after some large population-based studies to confirm the difference of their expression patterns, these miRNAs can be used as markers, which can differentiate between gastric cancerous and benign gastric ulcerous patients (33, 73, 74, 85, 86). 

It can be concluded that large numbers of miRNAs are closely related with GC in gastric juice, signifying that these miRNAs may have potential role in diagnosis of GC (87-89). With the help of microarray technology, it has been shown that a number of miRNAs are related with GC with noticeable expression changes, in which several of these miRNAs were significantly upregulated in GC endothelium as compared to the normal healthy endothelium; in the meantime other miRNAs such as mir-128b, mir-129 and mir-148 were reported to be downregulated in undifferentiated GC tissue (46, 89-91).

In past and current scenario, it can be concluded that some unique miRNAs are linked with the progression and prognosis of GC as significant prognostic markers, and the prominent miR-21 expression was significant when correlated with size and depth, but the low expression levels of miR-451 and miR-125a-5p was significantly linked with tumor size, tumor invasion, liver metastasis, and poor prognosis (89, 87, 92, 93). Downregulated miR-409-3p and miR-221 expression in patients could be prone to suffer from lymph node metastasis (46, 94, 75). In one of the multivariate analysis of miR-10b, miR-21, miR-223, miR-338, let-7a, miR-30a-5p and miR-126, it was concluded that the risk signatures can be autonomous predictors of the overall survival (95, 96) and the progression-linked signature of the miRNAs (like miR-125b, miR-199a, and miR-100), which were related with unfavorable outcomes in OS independent of clinical covariates such as depth of invasion, lymph-node metastasis and stages (89, 66). Moreover, extracellular miR-196a detected in conditioned medium was strongly correlated with its cellular expression status, and the increased circulating miR-196a in patient serum was associated with GC disease status and relapse (89, 97). 

Recent findings have shown that the best and specific cancer biomarkers for diagnosis and prognosis should be peripheral blood markers in order to make easy the health check-up of mass screening, follow ups of those who are at higher risk and also facilitate checking the disease at early and curable stage, which is only possible due to these non-invasive biomarkers. In 2008, three international research groups independently found that the miRNA are highly stable in peripheral blood of humans and other mammals nearly at the similar time and revealed that the expression status of particular peripheral blood miRNAs could make up a “molecular fingerprint” for diagnosis of cancers and other diseases (67-69, 89). Also in the past few years, it has become feasible to detect the serum concentration of miRNA in GC, which may be due to the progression of cancer to a more malignant phenotype or metastasis (98, 99). Recent studies have shown that the expression of some miRNAs was upregulated, (including miR-20b, miR-20a, miR-17, miR-106a, and miR-18a, miR-21 and miRNAs like miR-17-5p, miR-21, miR-106a and miR-106b) (50, 5). Next generation sequencing results confirmed that a panel of 19 serum miRNAs was clearly upregulated in GC patients compared to the controls, and five serum miRNAs among this panel (miR-1, miR-20a, miR-27a, miR-34 and miR-423-5p) was identified as a biomarker for GC detection; in this regard most such studies are required with large sample size and different types of cancers so that final clinical conclusion may be concluded regarding such cancer detection biomarkers. The results of a recent study as comparing pre- and post-operative blood plasma miRNA levels led to the detection of two miRNAs (miR-451 and miR-486) as potential GC detection biomarkers, as they were highly abundant in blood plasma and showed a marked decrease in post-operative plasma samples. So, further studies with large sample size and different types of cancer samples can help to prove that which particular sequence of miRNAs can act as biomarker for GC (89, 83, 79).

Till date, there are few main quantification methods for analysis of miRNAs such as relative quantification by a stem-loop reverse transcription PCR, microarrays and next-generation sequencing (73, 98). In current research field, the quantitative RT-PCR has been broadly used for the sensitive detection of low abundant circulating miRNAs with high accuracy and reproducibility (89, 100). And latest sequencing technologies could result in a step towards the increase in the rate of newly described microRNAs (89, 101). Furthermore due to the considerably variable results, better standardization methods are required in advanced research technologies. So far, several normalization strategies for the analysis of circulating miRNAs are available, especially housekeeping miRNAs (83).

Down-regulation of miRNAs by tumor suppressor utility may increase translation of oncogenes and therefore creation of overload oncogenic proteins, which may lead to tumor creation. Further upregulation of oncogenic miRNAs can block tumor suppressor genes and also lead to tumour creation

**Table-1:** List of microRNAs (miRNAs) as diagnostic/ prognostic biomarkers in plasma/serum/tissue/ markers for gastric cancer

**Diagnostic biomarkers plasma**			
**MiRNA**	**Sample**	**Method adopted**	**Ref.**
miR-199-3p	Plasma	qRT-PCR (real-time) miRNA microarray	**[** 67 **]** **[** 68 **]**
miR-20a, miR-106b and miR-221	Plasma	qRT-PCR (real-time)	**[** 69 **]**
miR-486 and miR-451	Plasma	miRNA microarray and qRT-PCR	**[** 70 **]**
miR-17-5p, miR-106a, miR-106b, miR-21, and let-7a	Plasma	qRT-PCR	**[** 71 **]**
miR-195-5p	Plasma	qPCR (real-time)	**[** 72 **]**
**Diagnostic biomarkers in serum**			
miR-21	Serum	qRT-PCR (miR-16)	**[** 73 **]**
miR-221, miR-376c and miR-744	Serum	Taq Man low-density array and Taq Man qRT-PCR	**[** 74 **]**
miR-378	Serum	miRNA microarray and qRT-PCR( real-time)	**[** 75 **]**
miR-195-5p let-7a	Serum	qRT-PCR ( real-time)	**[** 72 **]**
miR-196a	Serum	qRT-PCR ( real-time)	**[** 76 **]**
miR-451 and miR-486	Serum	Microarray analysis RT-PCR ( real-time)	**[** 77 **]**
**Diagnostic biomarkers in tissue**			
miR-223, miR-21 and miR-218	Tissue	qRT-PCR ( real-time)	**[** 78 **]**
miR-17-5p, miR-21, miR-106a, miR-106b and let-7a	Tissue	qRT-PCR ( real-time)	**[** 71 **]**
miR-195-5p let-7a MiRNA- 199a-3p	Tissue	qRT-PCR ( real-time)	**[** 80 **-** 81 **]**
**Prognostic biomarkers**			
miR-17-5p/20a, miR-21	Plasma	qRT-PCR ( real-time)	**[** 82 **]**

**The future prospects of miRNAs in gastric cancer**

One of the biggest advantages of using miRNA for therapeutic reasons would be because it can target multiple genes involved in a similar pathway (102). By targeting miRNAs that inhibit the normal functioning of the cell cycle, researchers are able to knock these proteins out to restore the regular functioning of cell cycle. In order to make miRNAs more successful in the realm of cancer therapeutics, scientists are currently involved to discover the ways to modify synthetic miRNAs for easier transfer to host cells in vivo. By altering certain structural elements such as the 2’-OH of ribose or phosphate backbone of synthetic miRNAs, it is less likely to succumb them to nuclease degradation. The cellular miRNAs are prone to nuclease degradation and their processing machinery tends to be insufficient, which lowers their bioavailability (77, 103). So, there is need of advanced ways to modify synthetic miRNAs by packaging the miRNAs in viral vectors, nanoparticles, or vectors containing tandem repeats of miRNAs (antisense sponges) without including host inflammatory responses, mutations of proto-oncogenes, cytotoxicity, and high cost (104). In addition, this modification should be in a way to minimize the risk of abnormal accumulation of miRNAs in the cells, which could overwhelm RNA-induced silencing complex (RISC) and cause major issues with the functions of normal miRNAs (105).

***Evidence of promise in the future of cancer prevention***

With a lot of recent information on miRNAs, there is evidence of promise in the future of GC prevention, prognoses, and therapeutics. However, there is still much work to be performed in this field, but progress is being made daily to understand how miRNAs work and how this can be applied to prevention of cancer. Not only miRNAs have the potential to serve as diagnostic and prognostic biomarkers, they can also be considered as biomarkers for predicting the response to therapy in several malignancies. Due to the specificity of miRNAs in tissue and disease stage specific expression, miRNA not only can identify the incidence or progression of tumors but they can also determine the primary organ or any specific tissue that is affected. In addition, they can identify the clinical and pathological stage of the disease. Furthermore, miRNAs can predict risk of progression, relapse, and metastasis, and help to evaluate possible clinical scenarios in relation to the therapy response. The clinical validity of these candidate miRNA signatures should be determined using large independent cohorts in multi-centric studies. In combination with the use of more robust platforms, more appropriate and accurate bio-computational and advanced statistical software should be introduced to analyse and identify candidate miRNA signatures. Much of the current literatures describing serum/plasma based miRNA expression profiles do not describe the sub-types of circulating miRNAs, suggesting that future large and different type of multiple cancerous studies concerning circulating miRNAs for diagnostic and prognostic purposes should focus on the type of circulating miRNAs present in body fluids (46, 89, 99, 103).

**Challenges during working with miRNAs**

(i): One of the biggest challenges for delivering miRNAs into tumor tissues is the fact that penetration of the miRNA (or miRNA mimic) into the tumor is rather inefficient (106), because the tumor’s leaky structure leads to inadequate blood perfusion (107). (ii): Another major challenge is that miRNAs are typically unstable and are degraded by nucleases in the blood when inserted into the body (108). (iii): In addition to these challenges, scientists also face with the problems of toxicity (as mentioned above), low uptake of miRNAs into cancer tissue (106) and off-target effects of miRNA delivery (109).

**How miRNAs can alter signaling in gastric cancers**

As per the earlier and recent research studies, gastric carcinogenesis is a multistep process involving the genetic and epigenetic alteration of protein-coding, proto-oncogenes and tumor-suppressor genes. But, advanced molecular biology added a new light on the involvement of a class of non-coding RNA known as miRNA in GC (110, 111). A considerable sum of miRNAs showed differential expression in tissues/serum/plasma of GC samples, it may be due to their oncogenic or tumor suppressor nature. Due to this nature, they may inhibit the expression of target genes, some of which are either directly or indirectly involved with canonical signaling pathways (111, 112). Over the years of studies, it becomes apparent that some miRNAs functionally integrate into multiple critical cell proliferation pathways, and dysregulation of these miRNAs is responsible for evading growth suppressors and sustaining proliferative signaling in cancer cells (111, 113, 114). The altering level of a single miRNA can trigger a cascade of signaling events culminating in a comprehensive increase or decrease in proliferation, apoptosis, cell growth etc. Successful miRNA targeting strategies could yield remarkable results, potentially altering the course of the disease. Understanding this will allow us to take a big step forward in the treatment of GC.

## Conclusion

The miRNAs that are specific to GC may act as ultimate early diagnostic and prognostic biomarkers for GC, due to involvement of these miRNAs in the progression of cancer. However, molecular biology of GC has been well characterized, but works on miRNAs in GC is still in its early stage. So, there is need of quantification and normalization strategies and standardization of the procedure before any novel miRNAs can act as a non-invasive marker for early diagnosis of GC.
